# The Challenges of Telemedicine in Rheumatology

**DOI:** 10.3389/fmed.2021.746219

**Published:** 2021-10-13

**Authors:** Yujie Song, Laurène Bernard, Christian Jorgensen, Gilles Dusfour, Yves-Marie Pers

**Affiliations:** ^1^IRMB, University of Montpellier, INSERM, CHU Montpellier, Montpellier, France; ^2^Clinical Immunology and Osteoarticular Diseases Therapeutic Unit, Department of Rheumatology, Lapeyronie University Hospital, Montpellier, France; ^3^IRMB, University of Montpellier, CARTIGEN, CHU de Montpellier, Montpellier, France

**Keywords:** e-health, telemedicine, rheumatic diseases, artificial intelligence, deep-learning

## Abstract

During the past 20 years, the development of telemedicine has accelerated due to the rapid advancement and implementation of more sophisticated connected technologies. In rheumatology, e-health interventions in the diagnosis, monitoring and mentoring of rheumatic diseases are applied in different forms: teleconsultation and telecommunications, mobile applications, mobile devices, digital therapy, and artificial intelligence or machine learning. Telemedicine offers several advantages, in particular by facilitating access to healthcare and providing personalized and continuous patient monitoring. However, some limitations remain to be solved, such as data security, legal problems, reimbursement method, accessibility, as well as the application of recommendations in the development of the tools.

## Introduction

Telemedicine is a form of remote medical practice based on the use of information and communication technologies. Its objective is to improve access to healthcare and the quality of life of patients by providing care and follow-up in their own residence, particularly for patients with chronic diseases, like those in rheumatology (1). Managements must therefore be employed to meet different needs: diagnosis, disease monitoring, therapeutic adaptation, or therapeutic education. The rapid evolution of technologies in recent years has forged forward the widespread development of more sophisticated connectivity allowing continuous and personalized services for patients.

Telemedicine has different possible applications in rheumatology, and its interventions are blooming and spreading. In this review, we will specify the different availabilities using tele-rheumatology, and also detail the benefits, limitations, and the perspectives of these technologies.

## Main Areas of Development for Telemedicine in Rheumatology

### Teleconsultation and Telecommunication

With the growth of speed and capacity in the geographical coverage of the internet network, telecommunication such as telephone, video, SMS, e-mail is in widespread use among remote consultations with the patient, but also discussions between specialists (tele-expertise). With the outbreak of the COVID-19, the switch to teleconsultation has become an urgent necessity. In India, Padmanabha et al. showed that teleconsultation in rheumatology during this pandemic was feasible with a high rate of satisfaction and prevented the discontinuation of medical follow-up for nearly three quarters of patients (2). Thus, regardless the diverse health system in each country, this particular health crisis period has considerably changed the traditional practices and rheumatologists should do their utmost to meet the needs of patients.

### Mobile Applications

Mobile applications (apps) represent an opportunity to improve health status and disease management by collecting large data, and play an important role particularly in peer support (patient-to-patient communication), which provides mutual exchange between patients in terms of knowledge, experience, and emotional, social or practical support. In rheumatology, there are nearly 200,000 available apps on Apple or Android devices, however, only a few have been rigorously evaluated and approved with clinical benefits (3). Patients with rheumatoid arthritis (RA) need regular monitoring by a rheumatologist to achieve good health outcomes, as a result, RA apps have become currently most demanding ones in mobile market (4), but recent studies assessing the quality of RA apps showed that most of them were not achieving high-quality scores and that data on funding and origin were frequently unavailable (5, 6). Therefore, the latest EULAR (European League against Rheumatism) recommendations emphasize the supervision of application development in patient and caregiver involvement, transparency, and accessibility (7). When it comes to the choice of apps, patients' preferences are for those with the capacity to inform them about biological results, treatments and disease activity. Besides, simple operation, therapeutic advice, useful information content, and the notifications like self-monitoring are also important factors need to be addressed (8).

### Wearable Technologies

Wearable devices are widespread tools. In 2017, 17% of adults used a connected watch or bracelet in the United States. These sensors passively collect a variety of data such as step count, heart rate, or sleep quality. Among them, inertial sensors specifically collect movement data. The study of articular cartilage, in particular cartilage-related pathologies, has reached a milestone where the fusion of data from imaging, biology and biomechanics for a better understanding of the pathological mechanisms. While imaging tools (CT, MRI) and biological tools are widely used in hospitals, biomechanical measurement technologies are often limited to laboratories. With the portable technologies in biomechanics are now largely available, the use of such metrological tools allows the extraction of biomechanical measurements more accessible and capable of following participants in their daily tasks and thus in so-called ecological movements. However, one of the difficulties is to develop the robust and understandable biomechanical markers that make sufficient sense for the improvement of rheumatic diseases.

**Figure 1:**
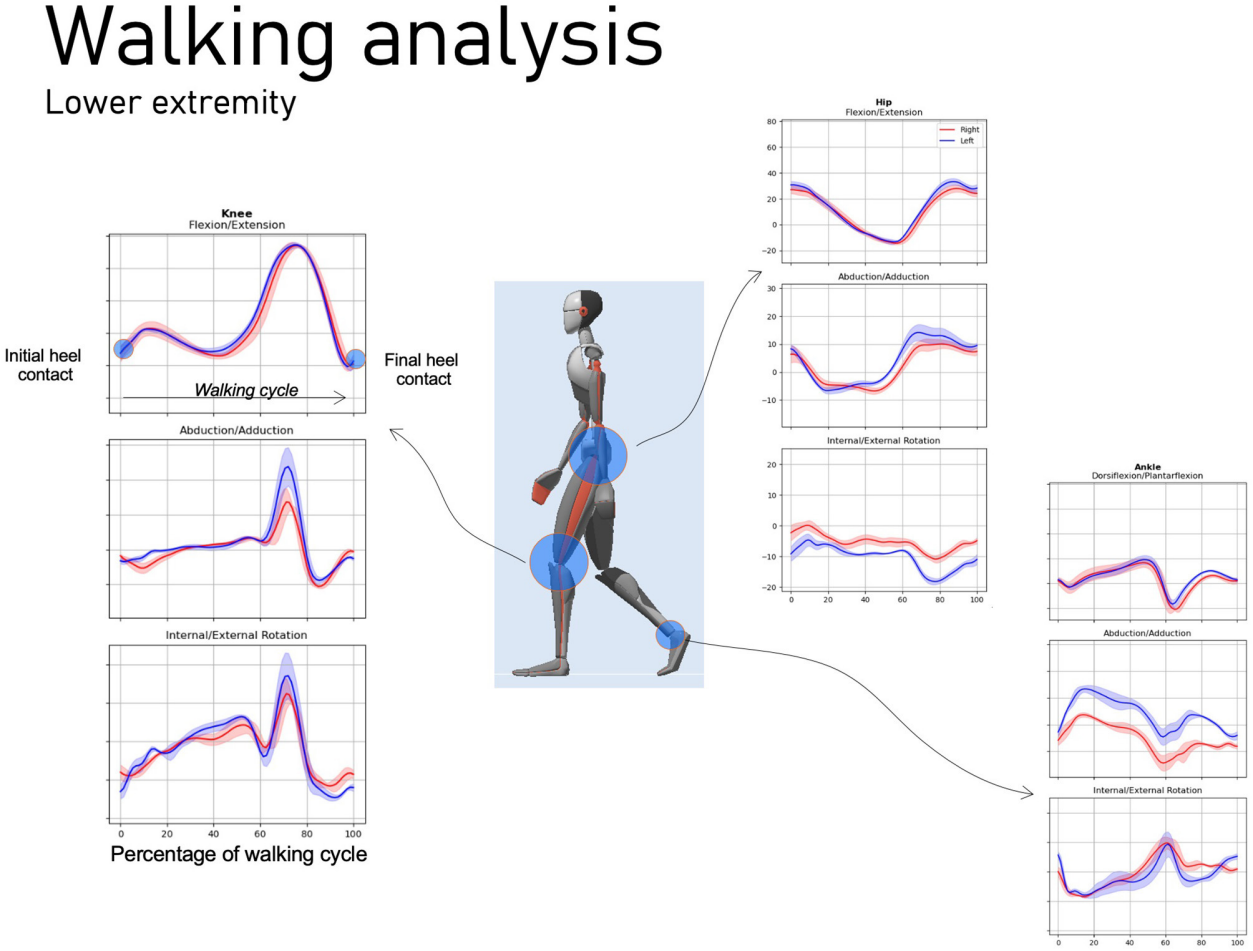
Evaluation of movement *via* inertial sensors. These devices enable to objectively analyze patients' movements and to develop diagnostic, prognostic or rehabilitation applications on rheumatic and musculoskeletal diseases.

By synchronously recording accelerations (actimeters) and angular velocities (gyroscope), a precise evaluation of the kinematics can be defined. Actimeters, as portable biomechanical sensors relevant to the study of RA, were first developed for daily cycles use, such as sleep/physical activity (9), but it is now possible to quantify the amount of functional movement and even to identify the type of movement performed, *via* artificial intelligence (AI) tools (10). This type of sensor has the advantage of using very little energy and can therefore be worn continuously for 10 days, which makes it possible to measure all the behavioral variability of patients. Further, Gossec et al. used a physical activity tracker to evaluate the association between flare-ups in RA and the impact on physical activity level. This tracker could allow early detection of disease flare-ups by observing changes in the number of steps (11). With regard to more precise kinematic measurements, in particular joints angles and spatio-temporal parameters like cadence, step length, and percent of mono-bipodal support, which used to be calculated in gait analysis laboratories, now it is possible to use inertial sensors (IMU). For example, Xsens combination (Figure 1), as a complex system but especially of inertial sensors (Xsens dot) and dedicated algorithms, could simplify the identification of these parameters (12). Connected insoles also seem a very promising tool for obtaining spatio-temporal and baropodometric parameters in ecological situations (13).

On the other hand, active sensors that allow us to perceive health states by questioning patients at a certain point regularly during the day would be a promising management to avoid the biases due to subjective factors in questionnaires. For example, we recently developed a dynamometer, linked to a smartphone, that allowed the patient with RA to self-assess the grip strength of the dominant hand. We observed an inverse correlation between the disease activity score (DAS28) and the handgrip strength exerted by the dominant hand (14). This device is an objective measure of RA activity and appears to be useful for monitoring patients at a distance. Nevertheless, these raw data must be transformed into digital biomarkers defined as numerical physiological and behavioral measures that explain, influent or predict health states. Thus, wearable devices could also actively intervene and constitute “digital therapeutics” (15).

### Digital Therapeutics

Simply monitoring collected data remotely does not generally lead to clinical improvement (16). Active interventions using “digital therapeutics” alone or in combination with conventional treatments are supposed to directly prevent, manage or treat certain pathologies (15). These interventions must prove their effectiveness, ensure data security and require validation by the authorities. To date, there are no approved devices for rheumatology, but some companies are looking into the development of technologies for pain management (17). Recently, we developed a connected interface (SATIE PR) operating with the help of a project manager to provide remote monitoring in patients with active RA initiating a new disease-modifying therapy. Our randomized controlled trial showed that disease control with the connected interface was similar to that of conventional follow-up and that the number of physical visits was significantly reduced in the connected group after 6 months (18). It was also the first time that a connected monitoring application showed a positive effect on quality of life in RA (18). Indeed, use of telemedicine may optimize disease monitoring allowing that face-to-face time can be dedicated to more severe or complex patients.

Moreover, the use of these different technologies should allow the identification of psychological and biomechanical profiles that show a good response to the different therapies tested. At the same time, these portables technologies allow for simplified longitudinal data collection and thus the identification of changes in the biomechanical behavior of the patient. This feature will allow continuous monitoring and identification of deviations. Also, the fusion of data from telemedicine and conventional clinical data may allow the identification of correlations between biomechanical markers and the quality of therapy (*via* X-ray, DAS28). This data fusion should also be promising to guide and anticipate management in order to optimize clinical treatment (19).

## Advantages and Limitations for Telemedicine in Rheumatology

### Benefits

Telemedicine offers different favorable applications in clinical practice. It helps to overcome the shortage of physicians, particularly in rural areas, to provide care outside of normal business hours, to save patient from travel, to facilitate services such as appointment scheduling and prescription renewals, and to meet economic constraints and users' expectations (20). In fact, patients with rheumatic diseases are very eager to use e-health technologies to better understand their chronic diseases (21). Patients also appreciate the ability to a more personalized care by selecting the outcomes that are most important to them, change and adapt their symptom monitoring as their disease progresses and their treatments change (22). Further, it is reported that regular monitoring of RA patients to detect disease flares improves outcomes, and a pilot study applying machine learning to activity tracker steps showed that physical activity was strongly linked to disease flares and that patterns of physical activity could be used to predict flares with great accuracy, with a sensitivity and specificity above 95% (23). In addition, patients and caregivers express a high level of satisfaction with the use of telemedicine without any complaints of loss of information compared to traditional consultations (24). In a review which evaluated the use of telemedicine in chronic pathologies, the data were heterogeneous but in cardiac and diabetic patients, telemedicine showed similar results to conventional medicine (25). In rheumatology, encouraging studies in tele-monitoring of RA have shown similar results in disease control compared to conventional monitoring (26, 27). In our study, we demonstrated an improvement in quality of life in patients benefiting from tele-monitoring (18) as well as a reduction in costs (unpublished data). Patients also feel reassured because they are provided with alerts and information, especially in case of infection. In addition, telemedicine creates a personalized support that enables continuous assistance to patients while participating in their therapeutic education and facilitating peer support. On the other hand, telemedicine based on the continuous measurement of physical data provides a paradigm switch in the evaluation and monitoring of certain pathologies. For example, actimeters offers a quantitative evaluation of movement in an ecological environment. It is then possible to monitor biomarkers in a daily and objective way under a natural circumstance of patients. Thus, actimeters could allow doctors to refine the appropriate treatments according to the collected biomarkers.

### Limitations

Although telemedicine can sometimes substitute a physical visit, some research paradoxically indicated that the use of telemedicine did not reduce the frequency of face-to-face consultations (28). It should also be emphasized that this remote service must not affect mutual trust between patient and doctor. In addition, initial evaluation of any rheumatic disease that needs detailed and thorough clinical evaluation, as well as some certain rheumatic diseases other than arthritic conditions, such as lupus, systemic sclerosis, and vasculitis, where direct patient to doctor communication is necessary for optimal understanding of the disease, deserves an indispensable place in the medical consultation. That being said, with the progress in terms of early detection and follow up of chronical stable disease, telemedicine cannot be applicable to all cases. Various other limiting factors have also been highlighted, as follows: the concern that medical data would not be protected, the inadequacy of the legal supervision, the uncertainty about the conditions of reimbursement, the difficulties of long-term follow-up or the poor understanding of patients due to their lack of medical knowledge (27). In fact, in the field of digital technology, there is a dual development between rapid technological advancement and its application, and the latter is often more complicated. The access to telemedicine must be generalized so that the majority of patients have no trouble using it, but also the system must not be vulnerable to protect patients. The recent EULAR recommendations should ensure that potentially uncontrolled and dangerous apps are ruled out and facilitate the development of safe tools by involving patients and doctors in their implementation (7). Last but not the least, the socioeconomic impact must also be taken into account, and studies evaluating cost-benefit analyses in medical specialties other than rheumatology have shown varied results (29). All the advantages and limitations of telemedicine are summarized in Table 1.

**Table 1:** The advantages and limitations of telemedicine in rheumatology.

**Advantages**	**Limitations**
a) Facilitating access to healthcare and to the rheumatologist	a) Personal data security needs to be improved
b) Improved communication i. Teleconsultation: patient-healthcare communication (rheumatologist, nurse.) ii. Tele-expertise: discussion between specialists	b) Legal supervision of the e-health field requires better definition
c) Enhanced control of rheumatic diseases and quality of life	c) Arrangement of the reimbursement method
d) Personalized and constant follow-up, and peer support	d) Lack of long-term follow-up
e) Availability of relevant medical information	e) Difficulty in understanding telemedicine devices due to insufficient medical knowledge
f) Strengthening therapeutic education and encouraging self-monitoring	f) Lack of access to the digital network and internet
g) Real-time monitoring of biomarkers using specific tools (actimeters, dynamometers, physical activity, etc.) and modification of treatments	g) Insufficient evaluation of the medical value and safety of available mobile applications
h) Satisfaction with the use of telemedicine devices	h) Lack of involvement of patients and doctors in their development
i) Reduction of health costs, especially transportation costs	i) Socio-economic effects are yet to be evaluated by specific studies

## Telemedicine Around the World

Despite the obvious benefits of telehealth, the actual adoption and uptake of telemedicine into mainstream practice worldwide has been slow. Bradford et al. identified that to reach the success and sustainability of telemedicine in rural and remote, Australia has to address six key factors, as follows: vision, ownership, adaptability, economics, efficiency and equipment (30). While Zobair et al. demonstrated that patient self-efficacy, telemedicine experience, enjoyment and prior-satisfaction significantly impacted patients' behavior on telemedicine in rural communities of Bangladesh (31). With regard to telemedicine in rural areas of Africa which is needed the most by the poorest of the poor, it is least likely to be provided because of inadequate infrastructure and high connectivity costs, as well as limited awareness of telemedicine by healthcare workers and the patient community, and lack of government will (32). In addition, Shenoy et al. reported that the absence of guidelines and of legal perspective regarding telemedicine in India might be the reason for its limited development (2). Luciano et al. showed that culture indirectly influences telemedicine adoption in the United States and Brazil through information policy. This means that before bringing in telemedicine, authorities must consider the culture of the country and its policies under which the telemedicine will function to ensure that there is a synergy between the two (33). Xu et al. found that 58.66% of the township health centers in rural China applied telemedicine in 2017, and this proportion was much higher in western China, with the central region following and the lowest in the eastern region. In each region, the prevalence of telemedicine adoption also tended to be higher in the less developed province (34). During COVID-19 pandemic, it was reported that continuity of care for patients with rheumatic disease could be guaranteed through telemedicine, mainly through telephone consultations, while adoption of other forms of telemedicine, such as asynchronous communication and video consultations was still low in the Netherlands (35).

## Perspectives

A variety of potential improvements have been suggested to address the challenges of implementing telemedicine. The political and economic policy, and the reimbursement system play a critical role in the development of the technology (20). Collaboration with health care organizations, such as health insurance, and mutual insurance companies, will help to assess costs and social acceptability and thus better manage reimbursement issues. The utilization of a shared design model in which patients contribute to the development of the apps, as well as the training of physicians in these new technologies, would facilitate the reliable integration of telemedicine into daily practice (20, 36). Moreover, it must be taken into account that the combination of a digital application assisted by a human approach makes the system more efficient (18). The digital assistant, by guiding patients using apps remotely, not only enables an optimization of the use of the technology but also to evaluate its limits and to connect it with the physical medical care when necessary. This capacity for human reflection is not yet within the range of technology, but research into the development of artificial intelligence is trying to get closer to it. Currently, the use in social networks to connect patients and assess health intelligence could be put to good use to differentiate the specific needs of vulnerable groups and provide personalized strategies (37).

### Artificial Intelligence and Machine Learning

Artificial intelligence (AI) is a branch of computer science that at tempts to both understand and build intelligent entities, often instantiated as software programs (38). While machine learning (ML) is a field of computer science that allows computer systems to learn and create predictive models from data, and makes use of algorithms, methods and processes to uncover latent associations within the data and to create descriptive, predictive or prescriptive tools that exploit those associations (38). Although there are no clear definitions or boundaries between AI and ML, and they often overlap, to our knowledge, AI is broader than ML in that it uses the latter as a prediction engine feeding decision support and recommendation systems that are more than the sum of their parts. In recent years, ML has gained much interest and been more and more accessible in medical fields, and its ultimate goal is to improve patient care and to facilitate clinical decision-making (39). With regard to rheumatology, AI or ML based on a range of data sources including clinical, biological and radiological data, has shown its potential and been applied in different aspects of these complex and heterogeneous diseases. For example, the evolution of image analysis with AI currently not only enables postural and kinematic estimations *via* cameras providing 2 or 3 dimensional images, but also prediction of progression of osteoarthritis (OA). It's reported that using MRI image date with AI, it's possible to stratify knee joints into different OA morphological phenotypes (40). Besides, there are studies reported to performance AI or ML technology to forecast future patient outcomes of RA, such as the mortality or the state of patients' activity, using electronic health record data and it's promising to be shared across hospitals with diverse patient populations (41, 42). However, to our latest knowledge, AI/ML hasn't been used in typical clinical rheumatology practice, mostly limited by the quality of the data upon which they are developed and used (17).

## Conclusion

**Figure 2:**
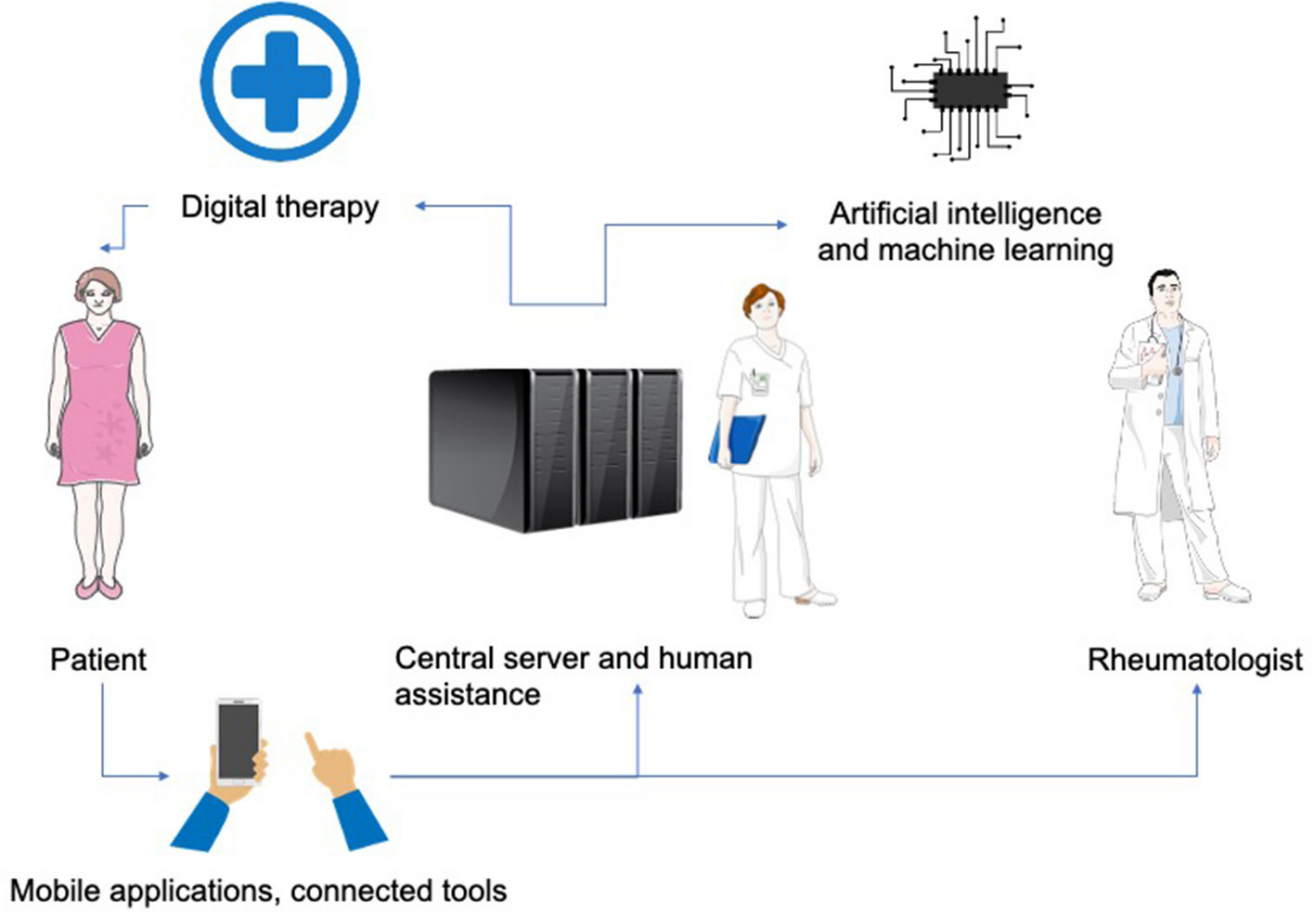
A general overview of telemedicine technologies integrated into medical practice in rheumatology. Patients upload data about their health status *via* mobile applications and online tools. This feedback is integrated into a central server and transmitted to the rheumatologist and other participants in the telemedicine system. Data analysis supported by human assistance and artificial intelligence assists rheumatologist in their medical practice (diagnosis, prognosis, therapy, follow-up) and provides patients with active assistance in the treatment and management of their pathology.

The utilization of digital technologies in healthcare would become an increasing trend in the future practice of rheumatology. The available devices are varied and can be integrated into everyday products offering a personalized and continuous approach (Figure 2). The explosion of telemedicine is also bringing new challenges for authorities, industries and doctors who must adapt to these innovative technologies and learn how to use them to maximize the patient's benefit.

## Author Contributions

CJ conceived the study design. Y-MP contributed to the planning and reporting of the work. LB drafted the first version of the paper in French. GD drafted one part of the paper about portable biomechanical sensors. YS drafted the final version of the paper that was revised and approved by all authors. All authors contributed to the article and approved the submitted version.

## Conflict of Interest

The authors declare that the research was conducted in the absence of any commercial or financial relationships that could be construed as a potential conflict of interest.

## Publisher's Note

All claims expressed in this article are solely those of the authors and do not necessarily represent those of their affiliated organizations, or those of the publisher, the editors and the reviewers. Any product that may be evaluated in this article, or claim that may be made by its manufacturer, is not guaranteed or endorsed by the publisher.
